# Electronic Cigarettes or Vaping: Are There Any Differences in the Profiles, Use and Perceptions between a Developed and a Developing Country?

**DOI:** 10.3390/ijerph19031673

**Published:** 2022-02-01

**Authors:** Muhammad Aziz Rahman, Bindu Joseph, Naima Nimmi

**Affiliations:** 1 School of Health, Federation University Australia, Berwick, VIC 3806, Australia; b.joseph@federation.edu.au (B.J.); dr.naimanimmi@gmail.com (N.N.); 2 Australian Institute for Primary Care and Ageing (AIPCA), La Trobe University, Melbourne, VIC 3086, Australia; 3 Department of Non-communicable Diseases, Bangladesh University of Health Sciences (BUHS), Dhaka 1216, Bangladesh; 4 Faculty of Public Health, Universitas Airlangga, Surabaya 60115, Indonesia

**Keywords:** e-cigarettes, vaping, harm reduction, nicotine, tobacco, Australia, Bangladesh

## Abstract

The use of electronic cigarettes or vaping is currently increasing in popularity globally. Debate continues regarding their potential role for smoking cessation. We aimed to compare the profiles, use and perceptions of using e-cigarettes amongst online forum users in a developed and a developing country. A cross-sectional survey was conducted among members of different popular online forums in Australia and Bangladesh who were current or ex-users of e-cigarettes. There were 422 study participants, 261 (62%) from Australia and 161 (38%) from Bangladesh. The mean age was 36.3 (±12) years and 83% were men. Australians were more likely to be exclusive users of e-cigarettes (70% vs. 30%, AOR 3.05 [95% CI 1.63–5.71]), but less likely to be dual users of smoking and e-cigarettes (43% vs. 57%, 0.36 [0.19–0.69]); they were also more likely to mention that the perceived reasons for using were their low cost, good taste/flavour, safety and assistance in reducing or quitting smoking (66% vs. 34%, 5.10 [2.04–12.8]), but less likely to mention a social/cool image as a reason for use (23% vs. 77%, 0.11 [0.01–0.87]) compared with Bangladeshi participants. About two-thirds of the participants in both countries perceived the use of e-cigarettes as less addictive than cigarettes and more than three-quarters perceived them as less harmful. E-cigarette users in Australia were more likely to use them to reduce or quit cigarettes compared with those in Bangladesh, and dual use was common in Bangladesh. These findings warrant the consideration of precautions for promoting e-cigarettes as a harm reduction strategy for smoking cessation in developing countries, such as Bangladesh.

## 1. Introduction

The use of electronic cigarettes (e-cigarettes), also known as vaping, is a modern form of smoking that has gained popularity globally in recent years [1]. These battery-powered devices vaporize a liquid solution containing nicotine and/or flavourings [2]. Although the long-term health effects are not known, the levels of certain toxic substances found in e-cigarettes are many times lower than those found in cigarettes [3]. The reasons for vaping are poorly understood; however, it appears that they are used both to quit the current smoking habit and to improve one’s social image [4].

Global debate continues regarding the effectiveness of vaping for smoking cessation. Several systematic reviews and meta-analyses indicated the effectiveness of vaping for smoking cessation or reduction in the number of cigarettes used [5,6,7], which is an important harm reduction strategy. A recent randomised controlled trial showed that vaping was more effective than nicotine replacement therapy (NRT) when accompanied by behavioural support [8]. Nicotine vaping was found to be more effective than non-nicotine vaping at aiding cessation [7]. A recent systematic review and meta-analysis also suggested that nicotine vaping was more effective than NRT and counselling alone, although the effectiveness decreased over time [9]. On the other hand, conflicting evidence was also found that did not support the effectiveness of vaping for smoking cessation [10].

Vaping has increased in popularity in Australia. The National Drug Strategy Household Survey (NDSHS) reported that the proportion of people who had ever used e-cigarette increased from 8.8% in 2016 to 11.3% in 2019, and the current use increased from 1.2% to 2.5% during that period [11]. In 2019, it was also reported that two in three (64%) current smokers and one in five (20%) non-smokers aged 18–24 have tried e-cigarettes; 4.1% of young people aged 14–24 years used e-cigarettes in Australia [12]. In the USA, it has increased particularly among middle and high school students and the prevalence of current use (in the last 30 days) was 13.1% (3.6 million students) in 2021, where the majority (80%) of current users reported using flavoured e-cigarettes [13]. Similar to this, other developed countries, such as Canada, New Zealand and the United Kingdom, also observed a higher rate of vaping [14].

The popularity and attractiveness of e-cigarettes have led to major concerns for public health implications nowadays [15]. Most of the e-cigarettes marketed globally contain nicotine; nicotine can be highly addictive and can have a damaging effect on adolescent brain development [16]. Unlike countries with more established markets for e-cigarettes, the commercial supply of nicotine for use in e-cigarettes in Australia is illegal (regulations differ across the states and territories) unless they are supplied through a doctor’s prescription [17].

E-cigarettes have also gained popularity in many developing countries. In Bangladesh, a developing country in South-East Asia, there has been an observed increased acceptance of e-cigarettes among the younger population because of the high impact from the Western culture [18]. However, there is a substantial lack of research on the use of e-cigarettes, particularly amongst younger people in Bangladesh. There are growing concerns among communities that e-cigarettes could be the initiator for future conventional cigarette smoking [19], although there is a disparity in the evidence on this issue. Individual behaviour of using e-cigarettes can be explained through the health belief model since such use is perceived as having low harm, as evidenced by pre-existing literature [20]. The health impacts of using e-cigarettes was reported by previous studies. Short-term use can cause respiratory irritation, repeated and prolonged central nervous system dysfunction and is a probable source of carcinogens for humans over long-term use, which was primarily evident through animal studies [21,22]. Bangladesh lacks data on the use of e-cigarettes and potential health impacts amidst the non-existence of regulations on using, advertising and promoting e-cigarettes there.

Tobacco control policies vary between Australia and Bangladesh, which may impact the use of e-cigarettes. While the nicotine delivery system has gained popularity in both Australia and Bangladesh, there has been very limited evidence from Bangladesh regarding the perceptions of use, the profiles of the users and use as a smoking cessation aid. Comparing these issues between Australia and Bangladesh would provide evidence to assist in future global tobacco control policies for e-cigarettes. Therefore, we aimed to compare the user profiles and perceptions of using e-cigarettes amongst the online forum users between Australia and Bangladesh as representations of a developed and a developing country, respectively. We would like to acknowledge the fact that during the period of conducting the study and publishing this paper, further developments occurred in terms of introducing new legislations around the purchase of e-cigarettes in Australia [17].

## 2. Materials and Methods

### 2.1. Study Design and Settings

This cross-sectional study included participants who were members of popular online forums of e-cigarette users in Australia and Bangladesh. The popular forums were identified through a Google search, which included online and Facebook forums, such as AussieVapers, Vaper Café Australia, E-cigarette Forum (Australia-New Zealand), Vape Central Western Australia, Vaping Australia, E-Cigarettes Australia, Vape Australia Buy and Sell and VapeCore from Australia, and Vapers Den Bangladesh, Vapers Hub Bangladesh and Vape Importers and Traders Association (VITAB) from Bangladesh. Electronic cigarettes, e-cigarettes and vaping were used as the key terms during the search. Data were collected from the study participants using a structured survey questionnaire via an online platform.

### 2.2. Study Population and Sampling

Members of the selected online forums were included as the study participants. To calculate the required sample size, at first, the total members/likes for each forum were identified; then, the presence of online members was monitored over a week and it was estimated that 10% of the members in Australia and 5% members in Bangladesh were active online members. The sample size was then calculated using OpenEpi [23] individually for each of the Australian and Bangladeshi forums. Considering a total of 1154 active online users in the Australian forums, the anticipated frequency of people using e-cigarettes within those online forums was 90%, 95% confidence intervals and 80% power, the estimated minimum sample size was 124. On the other hand, considering a total of 1597 active online users in the Bangladeshi forums, the anticipated frequency of people using e-cigarettes as 90%, 95% confidence intervals and 80% power, the estimated minimum sample size was 128.

### 2.3. Data Collection

Information about the study was sent to the administrators of those forums to obtain the necessary permission and the online survey followed the existing guidelines of internet research [24]. The survey was conducted in English in both countries. A structured questionnaire was developed based on the evidence from earlier studies, which used a validated measure for smoking and e-cigarette use [4,25,26]. An online survey link was developed with that questionnaire using Qualtrics from La Trobe University, Australia, and was posted on each of those forums. A reminder was also posted after four weeks and data collection continued for two months in 2017. The participant information sheet and the consent form were included in the first screen of the survey. Upon providing consent, participants moved to the next screen containing the full study questionnaire. Data were collected on socio-demographics; smoking and quitting behaviour regarding using cigarettes; use of e-cigarettes, including reasons to use, perceived health effects and addiction; and any other behavioural risk factors (such as alcohol use, physical activities, consumption of fruits and vegetables). There were 25 items in the questionnaire, which did not take more than 10 min to complete by a study participant. Necessary modifications in the language of the items were done following the pre-test of the questionnaire. Once the local ethics approval was obtained, data collection commenced and continued for the study duration. Data were collected anonymously so that no information that could identify any study participant was collected.

### 2.4. Data Analyses

All data from the Qualtrics platform were downloaded and data were analysed using STATA v.12. Descriptive analyses were used to describe the study variables. Inferential analyses were used to examine differences in the use and perceptions of e-cigarette users between Australia and Bangladesh. At first, chi-squared tests were used to determine the existence of association by comparing the categorical variables, and statistical significance was determined using *p* < 0.05. To examine the strength of associations, binary logistic regression was used and odds ratios (ORs), along with 95% confidence intervals (CIs), were calculated. Socio-demographic variables (gender, level of education and primary occupation) were considered as potential confounding variables and were adjusted for during multivariate analyses. This yielded adjusted ORs (AORs) and 95% CIs.

### 2.5. Ethics

Ethics approval was obtained from the Human Research Ethics Committee at La Trobe University, Melbourne, Australia (S17-095). As data were collected anonymously, it was not possible to withdraw from participation once the completed questionnaire was submitted online. However, the participants had the freedom to withdraw anytime during filling out the questionnaire online.

## 3. Results

A total of 422 people participated in this study. Most of the participants (261, 62%) were from Australia and 161 (38%) were from Bangladesh. The mean age (±SD) of the participants was 36.3 (±12) years and most of them (349, 83%) were males. More than half of the participants (129, 58%) from Australia were aged 31–50 years, whereas more than two-thirds (94, 69%) from Bangladesh were aged 18–30 years (*p* < 0.001). There was a significant difference (*p* < 0.001) in the sociodemographic characteristics of the study populations in Australia and Bangladesh (Table 1).

Current smoking of cigarettes (daily or occasional) was not common amongst study participants. Only a quarter of them (113, 27%) were current smokers, which was 18% (n = 46) in Australia and 41% (n = 67) in Bangladesh (*p* < 0.001). About three-quarters of the smokers (Australia: 74%, Bangladesh: 81%) had tried to quit smoking in the last 12 months; the most common strategy used for quitting attempts was cutting down (Australia: 83%, Bangladesh: 42%) (*p* < 0.001) (Table 2).

Almost all of the participants in Australia and Bangladesh were using e-cigarettes daily (98% vs. 88%, *p* < 0.001) and had nicotine in the e-liquid (92% vs. 98%). The average amount of e-liquid used, nicotine strengths and duration of use in Australia and Bangladesh were 9.1 (±7.6) vs. 6.4 (±7.3) mL/day, 7.5 (±11.1) vs. 4.3 (±2.0) mg/mL and 22.8 (±22.0) vs. 20.1 (±46.7) months, respectively (Table 3).

Table 4 shows the univariate and multivariate analyses results comparing users of e-cigarettes between Australia and Bangladesh. It was found that study participants from Australia were more likely to be exclusive users of e-cigarettes (70% vs. 30%, AOR 3.05, 95% CI 1.63–5.71, *p* < 0.001) and less likely to be dual users of cigarettes and e-cigarettes (43% vs. 57%, AOR 0.36, 95% CI 0.19–0.69, *p* < 0.01) compared with the Bangladeshi participants. Compared with the Bangladeshi participants, Australians were more likely to mention that the personal reasons for using e-cigarettes were the low cost (96% vs. 4%, AOR 59.6, 95% CI 20.9–170, *p* < 0.001), the good taste/flavour (71% vs. 29%, AOR 2.37, 95% CI 1.32–4.23, *p* < 0.01), they were safe to use (78% vs. 23%, AOR 2.83, 95% CI 1.54–5.19, *p* < 0.01) and to reduce/quit smoking (66% vs. 34%, AOR 5.10, 95% CI 2.04–12.8); they were less likely to mention the social/cool image aspect as a reason (23% vs. 77%, AOR 0.11, 95% CI 0.01–0.87, *p* < 0.05). However, there was no difference (*p* > 0.05) in the perceived addictiveness and health effects of using e-cigarettes, as well as the intention to quit in the next five years between Australia and Bangladesh.

## 4. Discussion

This study was one of the very few studies published so far that compared perceptions of using e-cigarettes between a developed and a developing country. In this study, the conveniently selected sample of Bangladeshi users was more likely to be younger (18–30 years), men, living in a metropolitan region, single, had undergraduate qualifications and an income source. While the selected sample for this study may not represent all Australians or Bangladeshis, we noted differences in a few issues. While exclusive use of e-cigarettes was common in the sample from Australia, dual use of cigarettes and e-cigarettes was common in the sample from Bangladesh. Perceived reasons for using were more likely to be related to cost, taste/flavour, safety and to reduce/quit cigarette smoking amongst the Australian participants; meanwhile, they were more likely to be related to the social/cool image amongst Bangladeshi counterparts. There was no difference in the perceptions of addictiveness or health effects or intention to quit vaping in the next 5 years amongst users of e-cigarettes in the samples from Australia and Bangladesh.

The sociodemographic profile of the e-cigarette users in Bangladesh who participated in this study was identified. More than two-thirds (69%) of the users in the Bangladesh sample were aged 18–30 years, while the majority of Australian users (47%) were aged 31–50 years. While there is a paucity of data from Bangladesh in general, studies investigating smoking behaviour amongst the university students of the Asia–Pacific region showed a higher prevalence of e-cigarettes use compared with Western countries [19]. The participant profile of the current study was also similar to the above study. The proportion of people who used e-cigarettes in Australia rose according to the recent national data [15]. However, there are no national data from Bangladesh to estimate the trends of using e-cigarettes. The only study identified from Bangladesh, which was a small-scale cross-sectional online survey among university students and reported in 2020 showed that 32% of the participants were familiar with e-cigarettes and had used them at least once in their lifetime, but that the majority (63%) were unaware of the harmful impacts of using e-cigarettes [18]. That study also reported that one-third of the female students also used e-cigarettes despite the unacceptable socio-cultural norms of Bangladesh. Findings from the sample of this study highlight the impact of the less regulated tobacco legislation in the country and e-cigarettes should be included within the national tobacco control laws to restrict the sales, marketing and use by minors [27]. Further research with a representative sample from Bangladesh could provide further evidence.

The dual use of cigarettes and e-cigarettes is a concern if the smokers do not quit and keep continuing both vaping and conventional cigarette smoking together. We found that dual use was common amongst 57% of the study participants from Bangladesh and 43% from Australia. Our confidence in these findings was supported by other studies that have noted similar variations across countries. The UK reported 30.5% dual users [28], whereas it was 85% amongst current e-cigarette users in Korea [29]. In the sample from Bangladesh, dual use was high, potentially due to the gap in regularity policies compared with Australia, where restrictive policies exist [15,18]. Dual use was associated with several adverse effects in a Korean study which found an increased risk of metabolic syndrome, distress, depression, unhealthy eating, obesity and greater nicotine dependence in dual users compared with cigarette-only smokers and never-smokers [29].

Perceived reasons for vaping were identified from the selected sample of this study, with a variation amongst Australian and Bangladeshi participants. In the current study, the Australian participants stated that the main reasons for using e-cigarettes were the low cost, safety, taste/flavour and smoking cessation; meanwhile, the Bangladesh counterparts perceived vaping as ‘cool’. The findings of this study are supported by a recent review article, which reported that the perceived benefits of using e-cigarettes were improved taste and smell, curiosity, safety and smoking cessation compared with conventional cigarette smoking [30]. Because of the similarity in sensory stimulation between the use of cigarettes and e-cigarettes, along with scientific evidence of reduced harm, e-cigarettes have become popular amongst smokers, who see them as a safer alternative to smoking cigarettes. The use of e-cigarettes as a smoking cessation aid was the predominant reason for use amongst Australian participants in this study, which was also supported by another study [31]. A critical finding of the above study, along with the prior evidence, indicated alignment towards the ‘harm reduction’ approach in Australian settings by facilitating the switch of the current smokers. However, such an approach would not be applicable in Bangladesh. The use of e-cigarettes for social/cool image reasons amongst the sample of Bangladeshi participants in this study was supported by an earlier study [18] and warrants urgent intervention to restrict access and supply, specifically among young people, as well as to promote health awareness amongst the Bangladeshi population, as evidence indicated the potential risk of non-smokers using vaping as a gateway to smoking could not be ignored [18]. While those findings had limited generalisability to all Australians or Bangladeshis, future research could focus on further exploration of perceptions amongst the representative sample from Bangladesh.

The perceived addictiveness and understanding of the health effects of using e-cigarettes were no different between the Australian and Bangladeshi participants in this study. In general, the use of e-cigarettes was perceived as less addictive than cigarettes in this study. A large-scale study in the USA amongst 8th-to-10th-grade students also showed that the addictiveness of e-cigarettes was perceived as being lower than for cigarette smoking [32]. While smoking cigarettes was perceived as addictive and harmful [33], the use of e-cigarettes was not perceived as such in Bangladesh [18]. Likewise, an Indian study amongst e-cigarette users across the eight largest metropolitan cities reported that most of the participants perceived it as less harmful than cigarette smoking, with minimal side effects [34]. Similarly, in this current study, more than two-thirds of the study participants from both Australia and Bangladesh perceived e-cigarettes as less harmful than cigarettes. Although numerous studies published so far supported this claim, it needs to be acknowledged that evidence on the long-term health effects is still unknown [35]. While e-cigarettes can be considered as one of the tools for quitting tobacco [36] and is comparatively more effective than conventional nicotine-replacement therapy [8], they cannot be considered completely risk free. Evidence indicated that it might work as a gateway to smoking and contribute to the entry to nicotine addiction [37,38]. Symptoms including headache, vomiting, jaw pain, throat irritation, difficulty in breathing, inflamed mouth, coughing, asthma, asthma exacerbation and depression were reported in prior studies [18]. While the use of e-cigarettes was considered safe to use by one-third of the participants in this study, a lack of understanding of the health impacts could be due to the mislabelled nicotine content in the products or e-liquid [39].

The intention to quit the use of e-cigarettes was similar between the Australian and Bangladeshi participants in this study. The majority of the study participants did not try quitting their use and less than a third had the intention to quit in the next 5 years. This finding contrasted with the finding from the USA, which showed that 50.2% of adolescents aged 12–17 years considered quitting in the next 30 days [40]. However, our study participants were adults. Many factors could influence the decision to quit, including the sociodemographic characteristics of users, availability of the products and regulation of vaping in a specific country [34,40].

The regulation of e-cigarettes differs between Australia and Bangladesh. The use of e-cigarettes became popular globally in the last decade, despite ongoing extensive debates on their therapeutic use and adverse effects. In 2019, Australia had around 300,000 users of e-cigarettes, where 70% of them reported using nicotine e-cigarettes [41,42]. The Thoracic Society of Australia and New Zealand (TSANZ) developed a position statement stating that e-cigarettes have adverse lung effects and should not be recommended as an effective and safe strategy for smoking cessation [43]. Similar position statements for taking precautionary steps for legalising e-cigarettes in the absence of long-term unknown health effects were also provided by other government and non-government agencies in Australia, such as the National Health and Medical Research Council (NHMRC), Cancer Council Australia, Heart Foundation, Australian Medical Association (AMA) and Public Health Association of Australia (PHAA) [44]. However, the Royal Australian and New Zealand College of Psychiatrists (RANZCP) supported the legalisation and regulation of e-cigarettes as harm reduction tools, specifically for people living with severe mental health conditions, who had a disproportionately higher prevalence of smoking compared to the general population [45]. Prior evidence suggested that nicotine e-cigarettes were more effective for smoking cessation compared with non-nicotine products [7,8]. However, the Royal Australian College of General Practitioners (RACGP) recommended that nicotine e-cigarettes would be a ‘reasonable intervention’ when combined with behavioural support for people who tried quitting with first-line therapy (pharmacotherapy and behavioural support) but failed, but were still motivated to quit smoking [46]. Australia implemented a new law on 1 October 2021 prohibiting the sale of nicotine vaping products and limiting their access to being prescription only [41]. On the other hand, the recent Global Adult Tobacco Survey (2017) indicated that 0.2% of Bangladeshi adults used e-cigarettes [47]. Availability was indicated as the primary reason for use in the previous study [18]. Currently, e-cigarettes are not illegal in Bangladesh; however, the Government of Bangladesh indicated plans for restricting these products [48]. A neighbouring country, namely, India, having the second-largest number of adult smokers in the world, banned the sale of e-cigarettes in 2019 [49], although the ban was either to improve public health or to support the traditional tobacco farmers (a political agenda could not be ascertained).

There were a few limitations of this study. The survey responses in this study were collected 4 years ago (2017) and the popularity of e-cigarettes has increased over that period. However, there was no significant change in the regulation of e-cigarettes in both countries that could impact the validity of the findings of this study. Social media platforms were used to recruit study participants, which limited the inclusion of e-cigarette users who did not use social media in both countries. Therefore, the findings of this study might be more generalisable to the social media users only, and it was also likely that only active users during the study period had responded to our survey, resulting in a selection bias. In addition, the findings of our study were limited to people who could access online platforms to respond to our survey questionnaire; hence, generalisability was limited to Internet-literate people. We must also acknowledge that the sample was selected conveniently; therefore, the risk of selection bias could not be ignored, and the study findings were not generalisable to either Australia or Bangladesh. However, the findings provided important insights and evidence to conduct further large-scale studies with representative samples. As the survey was conducted anonymously, it was not possible to identify more than one response from any individual. However, it seemed very unlikely that it happened as there was no incentive to complete the survey multiple times and the security settings of Qualtrics prevented multiple entries from the same IP address. However, the study had significant power to test the hypotheses as the required sample size was achieved in both countries. Finally, although there was a difference between the sample from Australia and Bangladesh in terms of the distribution of socio-demographic variables, all of those variables could not be included in the multivariate analyses in order to select the best fit model. Therefore, findings could potentially be confounded by the variables that could not be adjusted in this study, in particular, the age difference between the two samples.

## 5. Conclusions

This study identified and compared the profile and perceptions of a sample of e-cigarette users between a developed and a developing country. Dual use was more common in the sample from Bangladesh compared with Australia, which warrants further attention to examine and target the high-risk individuals of Bangladesh identified in this study. Due to the increased prevalence of e-cigarette use amongst young people in this study sample from Bangladesh, there is a potentially high risk of switching to regular cigarette smoking and undermining the tobacco control efforts there. Smokers in Australia need supportive strategies for accessing vaping for smoking cessation from the General Practitioners as a second-line of treatment following the recent regulation. On the other hand, precautions need to be there for not promoting e-cigarettes as a harm reduction tool for smoking cessation in Bangladesh due to the risk of more potential harms than benefits. Future research could be planned with a representative sample from both countries to investigate our findings further.

## Figures and Tables

**Table 1:** Characteristics of the study participants.

Characteristics	All Population	Australia	Bangladesh	*p*
	Total, N (%)	Total, n (%)	Total, n (%)	
Total participants	422	261	161	
Age (years)	361	224	137	
Mean (±SD)	36.3 (12.0)	41.3 (11.7)	28.1 (7.2)	
Age groups (years)	361	224	137	*<0.001*
18–30	134 (37.1)	40 (17.9)	94 (68.6)	
31–50	169 (46.8)	129 (57.6)	40 (29.2)	
>51	58 (16.1)	55 (24.6)	3 (2.2)	
Gender	419	259	160	*<0.001*
Men	349 (83.3)	191 (73.7)	158 (98.8)	
Women	70 (16.7)	68 (26.3)	2 (1.3)	
Location of residence	421	261	160	*<0.001*
Metropolitan/urban	329 (78.1)	177 (67.8)	152 (95.0)	
Regional/rural	92 (21.9)	84 (32.2)	8 (5.0)	
Marital status	421	260	161	*<0.001*
Single	153 (36.3)	59 (22.7)	94 (58.4)	
Married/partnered	248 (58.9)	182 (70.0)	66 (41.0)	
Divorced/widowed	20 (4.8)	19 (7.3)	1 (0.6)	
Level of education	422	261	161	*<0.001*
Primary or secondary school	106 (25.1)	93 (35.6)	13 (8.1)	
Undergraduate (certificate/diploma/bachelor’s)	255 (60.4)	152 (58.2)	103 (64.0)	
Postgraduate (master’s and above)	61 (14.5)	16 (6.1)	45 (28.0)	
Primary occupation	421	260	161	*<0.001*
Student	66 (15.7)	11 (4.2)	55 (34.2)	
Have an income source (job/business)	266 (63.2)	168 (64.6)	98 (60.9)	
Retired	34 (8.1)	34 (13.1)	0 (0)	
Not working	55 (13.1)	47 (18.1)	8 (5.0)	
Weekly income (average in AUD)	412	255	157	*<0.001*
≤500	185 (44.9)	72 (28.2)	113 (72.0)	
501–1000	100 (24.3)	72 (28.2)	28 (17.8)	
>1000	127 (30.8)	111 (43.5)	16 (10.2)	
Behavioural factors (multiple responses)	422	261	161	
Drinks alcohol	76 (18.0)	61 (23.4)	15 (9.3)	*<0.001*
Performs less physical activity	156 (37.0)	86 (33.0)	70 (43.5)	*0.030*
Consumes less fruits and vegetables	151 (35.8)	82 (31.4)	69 (42.9)	*0.017*

*The p values in Italics indicate statistical significance (p < 0.05)*.

**Table 2:** Smoking behaviour of the study participants.

Characteristics	All Population	Australia	Bangladesh	*p*
	Total, N (%)	Total, n (%)	Total, n (%)	
Total participants	422	261	161	
Smoking cigarettes in last 30 days	421	260	161	*<0.001*
None	308 (73.2)	214 (82.3)	94 (58.4)	
Daily	46 (10.9)	19 (7.3)	27 (16.8)	
Not daily/occasional	67 (15.9)	27 (10.4)	40 (24.8)	
Current smokers				
Number of cigarettes smoked in last 30 days	107	45	62	0.412
1 or less	58 (54.2)	24 (53.3)	34 (54.8)	
2 to 9	33 (30.8)	12 (26.7)	21 (33.9)	
10 or more	16 (15.0)	9 (20.0)	7 (11.3)	
Duration of smoking cigarettes (years)	93	38	55	*<0.001*
Mean (±SD)	13.0 (9.1)	18.3 (9.7)	9.4 (6.7)	
Tried to quit smoking cigarettes in the last 12 months	108	46	62	0.098
No	24 (22.2)	12 (26.1)	12 (19.4)	
Yes, tried 1–10 times	71 (65.7)	32 (69.6)	39 (62.9)	
Yes, tried >10 times	13 (12.0)	2 (4.3)	11 (17.7)	
Ex-smokers				
Stopped smoking cigarettes	308	214	94	*<0.001*
1–6 months ago	99 (32.1)	65 (30.4)	34 (36.2)	
7–12 months ago	58 (18.8)	37 (17.3)	21 (22.3)	
13–60 months ago	117 (38.0)	88 (41.1)	29 (30.9)	
>60 months ago	27 (8.8)	24 (11.2)	3 (3.2)	
Never smoked	7 (2.3)	0 (0)	7 (7.4)	
Duration of smoking cigarettes (years)	282	201	81	*<0.001*
Mean (±SD)	21.0 (11.9)	24.6 (11.5)	12.0 (7.3)	
Strategies used in the attempt to quit smoking cigarettes (multiple responses)	415	261	154	
None	76 (18.3)	12 (4.6)	64 (41.6)	*<0.001*
Nicotine replacement products (e.g., gum, lozenge, mouth spray)	244 (58.8)	218 (83.5)	26 (16.9)	*<0.001*
Quitting medication (e.g., champix, zyban)	134 (32.3)	131 (50.2)	3 (1.9)	*<0.001*
Cold turkey (quitting abruptly)	249 (60.0)	221 (84.7)	28 (18.2)	*<0.001*
Cutting down (reducing the number/frequency)	281 (67.7)	216 (82.8)	65 (42.2)	*<0.001*
Counselling from professionals	84 (20.2)	77 (29.5)	7 (4.5)	*<0.001*

*The p values in Italics indicate statistical significance (p < 0.05)*.

**Table 3:** Use of electronic cigarettes or vaping among the study participants.

Characteristics	All Population	Australia	Bangladesh	*p*
	Total, N (%)	Total, n (%)	Total, n (%)	
Total participants	422	261	161	
Using e-cigarettes in last 30 days	416	261	155	*<0.001*
None	5 (1.2)	2 (0.8)	3 (1.9)	
Daily	392 (94.2)	255 (97.7)	137 (88.4)	
Not daily/occasional	19 (4.6)	4 (1.5)	15 (9.7)	
Amount of e-liquid/juice used per day (mL/day)	156	102	54	*0.034*
Mean (±SD)	8.2 (7.6)	9.1 (7.6)	6.4 (7.3)	
Nicotine in e-liquid/juice	280	199	81	0.085
No	18 (6.4)	16 (8.0)	2 (2.5)	
Yes	262 (93.6)	183 (92.0)	79 (97.5)	
Nicotine strength in e-liquid/juice (mg/mL)	142	92	50	0.050
Mean (±SD)	6.4 (9.1)	7.5 (11.1)	4.3 (2.0)	
Duration of using e-cigarettes (months)	337	221	116	0.457
Mean (±SD)	21.9 (32.6)	22.8 (22.0)	20.1 (46.7)	
Frequency of use per day	243	137	106	*0.002*
Mean (±SD)	23.8 (32.1)	29.4 (35.9)	16.5 (24.8)	
Reasons for using e-cigarettes (multiple responses)	411	259	152	
Low cost	173 (42.1)	166 (64.1)	7 (4.6)	*<0.001*
Good taste/flavour	211 (51.3)	149 (57.1)	62 (40.8)	*0.001*
Safe to use	160 (38.9)	124 (47.5)	36 (23.7)	*<0.001*
Can be used indoors/in smokefree areas	131 (31.9)	82 (31.7)	49 (32.2)	0.904
Social/cool image	13 (3.2)	3 (1.2)	10 (6.6)	*0.002*
To reduce/quit cigarette smoking	374 (91.0)	246 (95.0)	128 (84.2)	*<0.001*
Others	31 (7.5)	27 (10.4)	4 (2.6)	*0.004*
Ever tried quitting e-cigarettes	410	258	152	*0.003*
No	360 (87.8)	22 (8.5)	28 (18.4)	
Yes	50 (12.2)	236 (91.5)	124 (81.6)	
Perceived addictiveness of e-cigarettes	409	257	152	*<0.001*
Not addictive	106 (25.9)	73 (28.4)	33 (21.7)	
As addictive as cigarettes	33 (8.1)	9 (3.5)	24 (15.8)	
Less addictive than cigarettes	270 (66.0)	175 (68.1)	95 (62.5)	
Intend to stop using e-cigarettes in the next 5 years	410	258	152	*0.001*
No	88 (21.5)	81 (31.4)	35 (23.0)	
Maybe	206 (50.2)	112 (43.4)	94 (61.8)	
Yes	116 (28.3)	65 (25.2)	23 (15.1)	
Perceived health effects of using e-cigarettes	410	258	152	0.282
No harmful health effects	79 (19.3)	45 (17.4)	34 (22.4)	
Less harmful than cigarettes	329 (80.2)	212 (82.2)	117 (77.0)	
Same harmful as cigarettes	1 (0.2)	1 (0.4)	0 (0)	
More harmful than cigarettes	1 (0.2)	0 (0)	1 (0.7)	

*The p values in Italics indicate statistical significance (p < 0.05)*.

**Table 4:** Comparing the use of electronic cigarettes or vaping between the study participants in Australia and Bangladesh.

Characteristics	Total, N	Australia	Bangladesh	Unadjusted Analyses			Adjusted Analyses *		
		Total, n (%)	Total, n (%)	*p*	OR	95% CI	*p*	AOR	95% CI
Total participants	422	261	161						
Smoking behaviour	422	261	161						
Not used cigarettes or e-cigarettes	5	2 (40.0)	3 (60.0)	0.327	0.41	0.07–2.46	0.853	0.79	0.07–9.59
Only used cigarettes (exclusive users of cigarettes)	6	0 (0)	6 (100)	NA	NA	NA	NA	NA	NA
Only used e-cigarettes (exclusive users of e-cigarettes)	304	213 (70.1)	91 (29.9)	*<0.001*	*3.41*	*2.19–5.31*	*<0.001*	*3.05*	*1.63–5.71*
Used both cigarettes and e-cigarettes (dual users)	107	46 (43.0)	61 (57.0)	*<0.001*	*0.35*	*0.22–0.55*	*0.002*	*0.36*	*0.19–0.69*
Nicotine in e-liquid/juice	280	199	81						
No	18	16 (88.9)	2 (11.1)		1			1	
Yes	262	183 (69.8)	79 (30.2)	0.104	0.29	0.07–1.29	0.939	1.08	0.15–7.54
Reasons for using e-cigarettes (multiple responses)	411	259	152						
Low cost	173	166 (96.0)	7 (4.0)	*<0.001*	*36.8*	*16.6–81.9*	*<0.001*	*59.6*	*20.9–170*
Good taste/flavour	211	149 (70.6)	62 (29.4)	*0.001*	*1.95*	*1.30–2.93*	*0.004*	*2.37*	*1.32–4.23*
Safe to use	160	124 (77.5)	36 (22.5)	*<0.001*	*2.96*	*1.89–4.62*	*0.001*	*2.83*	*1.54–5.19*
Can be used indoors/in smokefree areas	131	82 (62.6)	49 (37.4)	0.904	0.97	0.63–1.50	0.319	1.37	0.74–2.53
Social/cool image	13	3 (23.1)	10 (76.9)	*0.007*	*0.17*	*0.05–0.61*	*0.037*	*0.11*	*0.01–0.87*
To reduce/quit cigarette smoking	374	246 (65.8)	128 (34.2)	*<0.001*	*3.43*	*1.72–6.83*	*0.001*	*5.10*	*2.04–12.8*
Perceived addictiveness of e-cigarettes	409	257	152						
Not addictive	33	9 (27.3)	24 (72.7)	0.136	1.43	0.89–2.29	0.712	0.88	0.46–1.70
Less addictive than cigarettes	270	175 (64.8)	95 (35.2)	0.249	1.28	0.84–1.95	0.234	1.43	0.79–2.60
As addictive as cigarettes	106	73 (68.9)	33 (31.1)	*<0.001*	*0.19*	*0.09–0.43*	0.164	0.48	0.17–1.35
Perceived health effects of using e-cigarettes	410	258	152						
No harmful health effects	79	45 (57.0)	34 (43.0)	0.223	0.73	0.45–1.21	0.074	0.52	0.25–1.07
Less harmful than cigarettes	329	212 (64.4)	117 (35.6)	0.203	1.38	0.84–2.26	0.059	1.99	0.97–4.08
Same harmful as cigarettes	1	1 (100)	0 (0)	NA	NA	NA	NA	NA	NA
More harmful than cigarettes	1	0 (0)	1 (100)	NA	NA	NA	NA	NA	NA
Intend to stop using e-cigarettes in the next five years	410	258	152						
No	88	65 (73.9)	23 (26.1)		1			1	
Yes	322	193 (59.9)	129 (40.1)	*0.018*	*0.53*	*0.31–0.90*	0.152	0.59	0.28–1.22

OR: odds ratio, AOR: adjusted odds ratio, 95% CI: 95% confidence interval, NA: Not Available due to small or nil cell counts; * Adjusted for: gender, level of education and primary occupation. The values in Italics indicate statistical significance.

## Data Availability

The data are available upon reasonable request from the corresponding author.
